# *In Vivo* Characterization of the Homing Endonuclease within the *polB* Gene in the Halophilic Archaeon *Haloferax volcanii*

**DOI:** 10.1371/journal.pone.0015833

**Published:** 2011-01-20

**Authors:** Adit Naor, Rona Lazary, Adi Barzel, R. Thane Papke, Uri Gophna

**Affiliations:** 1 Department of Molecular Microbiology and Biotechnology, George S. Wise Faculty of Life Sciences, Tel Aviv University, Tel Aviv, Israel; 2 Department of Molecular Cell Biology, University of Connecticut, Storrs, Connecticut, United States of America; Tulane University Health Sciences Center, United States of America

## Abstract

Inteins are parasitic genetic elements, analogous to introns that excise themselves at the protein level by self-splicing, allowing the formation of functional non-disrupted proteins. Many inteins contain a homing endonuclease (HEN) gene, and rely on its activity for horizontal propagation. In the halophilic archaeon, *Haloferax volcanii*, the gene encoding DNA polymerase B (*polB*) contains an intein with an annotated but uncharacterized HEN. Here we examine the activity of the *polB* HEN *in vivo*, within its natural archaeal host. We show that this HEN is highly active, and able to insert the intein into both a chromosomal target and an extra-chromosomal plasmid target, by gene conversion. We also demonstrate that the frequency of its incorporation depends on the length of the flanking homologous sequences around the target site, reflecting its dependence on the homologous recombination machinery. Although several evolutionary models predict that the presence of an intein involves a change in the fitness of the host organism, our results show that a strain deleted for the intein sequence shows no significant changes in growth rate compared to the wild type.

## Introduction

Inteins are parasitic genetic elements within open reading frames able to perform self-splicing at the level of the protein. The intein is transcribed and translated along with the gene in which it resides, and is subsequently excised from the protein between its two bordering exteins by an autocatalytic process, in which the exteins are joined together [1], [2]. Homing Endonucleases (HENs) are a diverse class of site-specific DNases found in archaea, bacteria and lower eukaryotes, and in some of their respective viruses [3], [4]. HENs are selfish genetic elements that reside within self splicing introns and inteins, and promote the horizontal propagation of their respective intron/intein into intron-less or intein-less alleles by cleaving the vacant target site to induce homologous recombination or reverse transcription. HENs recognize relatively long target sequences (14–40 bp), a fact that has made them a potential tool for gene therapy and genetic engineering [5].

**Figure 1:**
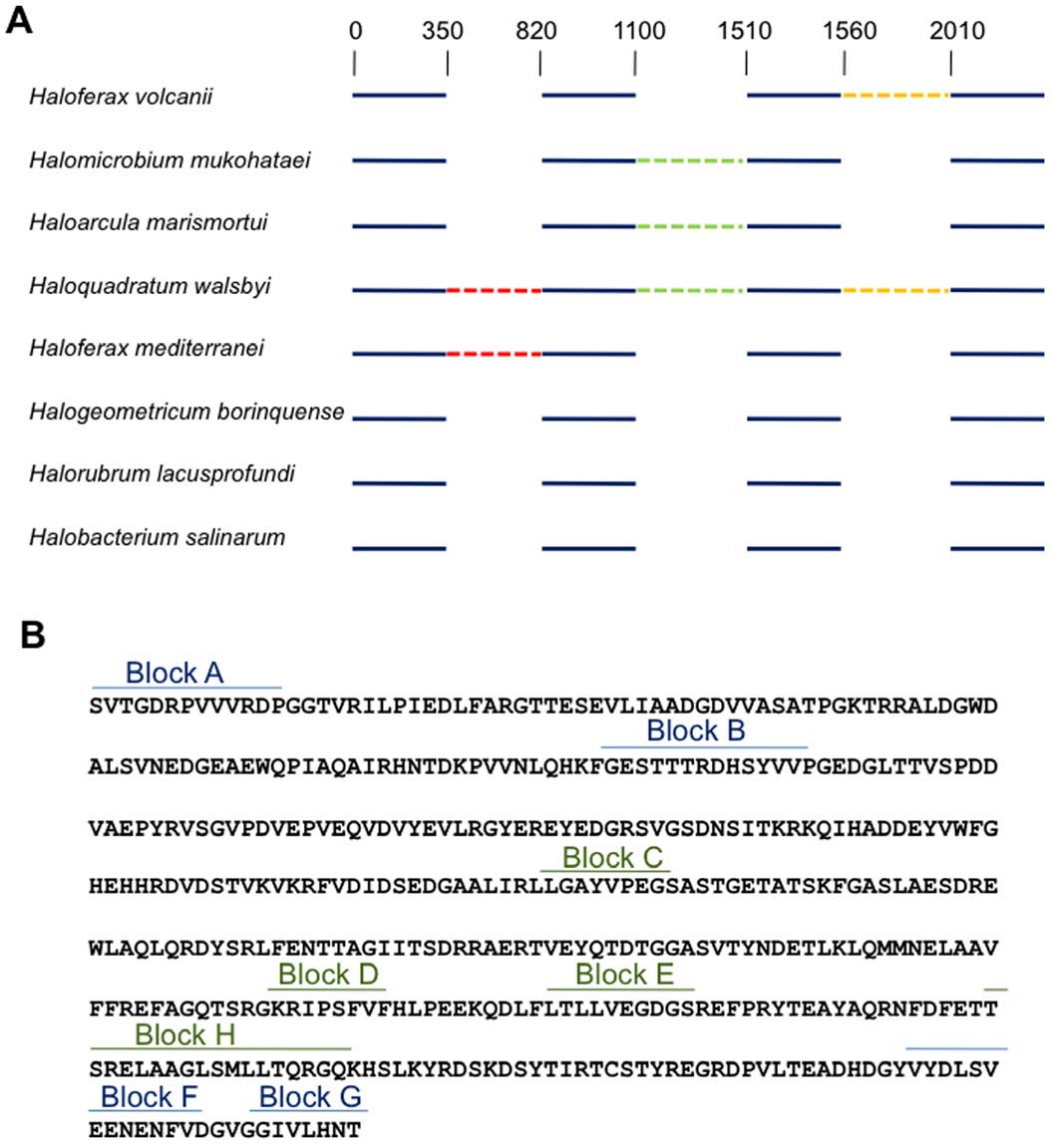
A schematic representation of the the *polB* gene. **A**. Several species of halophilic archaea (dotted lines represent intein sequences). **B**. *Haloferax volcanii polB* intein (blue: intein splicing motifs; green: HEN motifs).

The gene for DNA polymerase B is a known target for inteins in halophilic archaea [see InBase, the database of known inteins: http://tools.neb.com/inbase/index.php, [6]]. A multiple alignment of haloarchaeal *polB* homologs (Fig. 1A) revealed that three sites within these genes can contain intein insertions, and that at least one organism (*Haloquadratum walsbyi*) has inteins occupying all three locations. In *Haloferax volcanii* the *polB* gene contains a single 437 amino acid-long intein (Hvo PolB) inserted at amino acid position 1063 from the N-terminus, which has been annotated in InBase as having a putative HEN. It has been proposed that the presence of an intein involves a change in the fitness of the host organism [1], but this has not been tested experimentally. Here we assayed the *in vivo* endonuclease activity encoded by the HEN located in the *Hfx. volcanii polB* gene. We also generated a strain that was cured of the *polB* intein and tested its fitness.

## Results and Discussion

The *polB* gene of *Hfx. volcanii* is annotated in InBase as containing a putative intein with an endonuclease of the DOD (dodecapeptide) family. However, the only selfish element motifs previously recognized in this gene are the ones defining the intein, namely blocks A, B (characterizing the N-terminal protein splicing region), F and G (characterizing the C-terminal protein splicing region). In contrast, the blocks indicating the conserved domains in the HEN were not annotated. By aligning the amino acid sequence of Hvo PolB to that of known DOD HENs in InBase, we identified motifs corresponding to DOD blocks C, D, E and H, (see Figure 1B and Figure S1). This demonstrated that the Hvo PolB contains a DOD HEN that may be studied *in vivo*.

### Curing the intein is hampered by HEN activity

**Figure 2:**
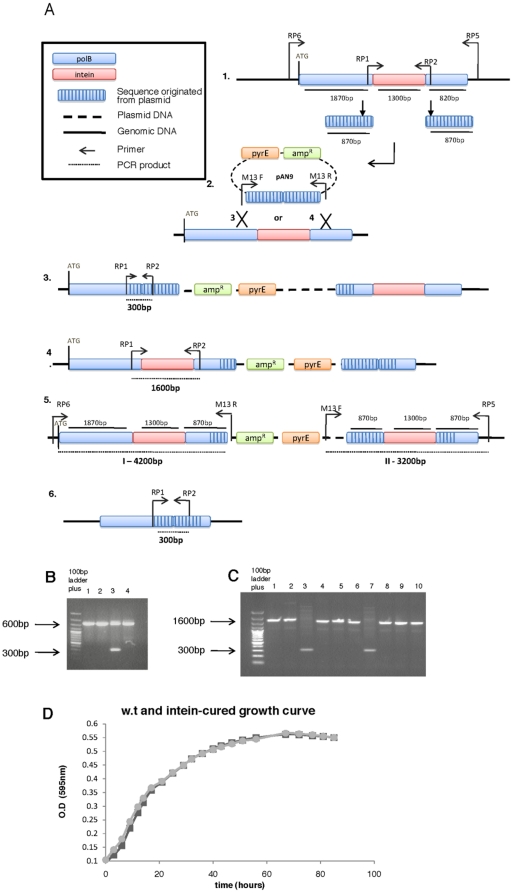
The *polB* ‘pop-in’/‘pop-out’ experiment. **A**. 1. The genomic region containing the w.t. *polB* sequence, indicating the fragments amplified and cloned to create pAN9. Arrows indicate primer binding sites. 2. The suicide vector pAN9, which contains 1700bp of the *polB* gene, without the intein. Striped boxes indicates sequence originating from the plasmid. Arrows indicate primer binding sites. 3+4. Two alternative expected ‘pop-in’ arrangements, following selection for plasmid integration. The integration of the plasmid is forced by selecting for *ura^+^* colonies. The plasmid can integrate, by a single homologous recombination event either by the region 5′ to the intein – resulting in arrangement 3, or through the 5′ region resulting in arrangement 4. 5. The ‘pop-in’ obtained in this experiment, in 7 out of 8 ‘pop-in’ colonies examined. I and II: two different PCR products (see figure 2B). 6. The desired ‘pop-out’ state. **B**. Agarose gel electrophoresis of PCR amplicons obtained from intein ‘pop-in’ candidates, using primers RP1 and RP2. Lane 1 – wild type, lane 2 and 4 – ‘pop in’ with an intein duplication see figure 2 A #3,4. Lane 3 – ‘expected ‘pop-in’, see figure 2 A #5. **C**. Agarose gel electrophoresis of PCR amplicons obtained from intein ‘pop-out’ candidates, using primers RP1 and RP2, see figure 2 A #6. Lane1 –w.t. cells; lanes 2,4,5,6,8,9,10 – ‘pop out’ back to the w.t. state; lanes 3 and 7 – deletion of the intein. **D**. A growth curve comparing the wild type WR532 to its intein-cured derivative.

Although inteins are present in many essential genes in numerous organisms, their potential effect on host fitness has not been tested [1]. To determine whether the presence of an intein in the *polB* gene of *Hfx. volcanii* affects the fitness of this archaeon, we attempted to cure the *Hfx. volcanii polB* gene of its intein. By employing the ‘pop-in/pop-out’ strategy for allele exchange, previously developed for *Hfx. volcanii* ([7], see materials and methods and figure 2), a plasmid construct was generated containing a *polB* gene fragment (approximately 1700bp out of about 4000bp) that includes the original stop codon at the 3′ end but not the intein (Figure 2A#1). Thus, an intein-less *polB* allele was created lacking the first 1000 nucleotides of this gene. The intein-less construct was created by overlap PCR (see materials and methods), cloned into the pTA131 vector [8], and the resulting suicide plasmid (pAN9, see Table 1 and Figure 2A#2), was transformed into the uracil auxotroph *Hfx. volcanii* strain WR532 (*ΔpyrE*). Transformed colonies were selected for on a medium lacking uracil.

**Table 1:** Plasmids used in this study.

Plasmid	Description	Primers used for the construction	Source or reference
pTA131	pBluescript II containing the *Hfx. volcanii pyrE2* gene- used for ‘pop-in’ ‘pop-out’ experiments		[8]
pAN9- pTA131 intein deletion	*Hfx. volcanii polB intein* flanking regions cloned into pTA131.	AP58,AP59, AP60,AP61	This study
pTA354	*E. coli/Hfx. volcanii* shuttle vector with *pyrE2* marker. Contains 948-bp *BmgBI-EcoRV* fragment of pTA250 with pHV1/4 replication origin inserted at *PciI* site.		[18]
pRL1	850 bp flanking regions on each side of the HEN recognition site/intein insertion site, cloned into pTA 354	The insert cut from pAN9	This study
pRL2	500 bp flanking regions on each side of the HEN recognition site/intein insertion site, cloned into pTA 354	RP7, RP8	This study
pRL3	250 bp flanking regions on each side of the HEN recognition site/intein insertion site, cloned into pTA 354		This study
pRL4	850 bp flanking regions on each side of the HEN recognition site/intein insertion site, with an altered HEN recognition site.	RP1–12	This study
pGEM-T-easy			Promega

The plasmid integration via homologous recombination occurs at either flanking region (Figure 2A#2), resulting in two possible different arrangements. In the first alternative, integration occurs through homologous recombination in the region 5′ to the intein, (Figure 2A#3) resulting in an intact *polB* gene lacking the intein, and a second copy containing only two 850 bp sequences surrounding the intein sequence. The second alternative, is that integration occurs 3′ to the intein (Figure 2A #4), and results in an intact, intein-containing, *polB* sequence, followed by a second sequence, containing only 850 bp flanking the intein. In both cases one intact *polB* gene will be expressed, but one version will express a cured *polB* while the other will produce an intein-containing PolB, including its endogenous HEN, which will later be excised and might be active.

The uracil prototrophs of WR532 obtained after transformation were screened by PCR using primers from both sides of the intein (RP1 and RP2, see figure 2A #6 and Table S1). PCR was expected to yield, for each colony, two different-sized amplicons, regardless of the integration site: one copy containing an intein, (a larger PCR fragment), and the second, originating from the plasmid, harboring no intein, thus producing a smaller PCR band (Figure 2A#3–4). Instead, seven out of eight colonies yielded only one band, corresponding to the w.t. length (about 1600bp), indicating that the intein sequence was present in both locations (Figure 2A#5). This observation was validated by two additional PCR reactions, using different primer sets. In each of these reactions one primer matched the chromosome, and the other the integrated plasmid (primers RP6+M13R and RP5+M13F, see Figure 2A#5). These PCR reactions resulted in amplicons sized 4200 bp and 3200 bp, respectively, as expected if both *polB* alleles contained the intein. The two PCR fragments obtained, (see figure S2 B) were cloned into a pGEM-T Easy vector and the inserts were fully sequenced (the primers used are listed in Table S1). DNA sequencing confirmed that in the ‘pop-in’ state (Figure 2A#5) two copies of *polB* were indeed present, one carrying the full gene, and the other lacking the first 1000bp. Strikingly, both copies carried the intein. These results indicate that gene conversion had occurred, probably mediated by the specific activity of the homing endonuclease.

In one of eight ‘pop-in’ colonies, the expected two PCR bands were obtained. This colony had the ‘pop-in’ arrangement illustrated in figure 2A#3 (verified by PCR using primers RP4 and RP2, see figure S2 C). This arrangement results in a HEN gene that should not be transcribed, because it is no longer part of the PolB open reading frame and lacks a promoter sequence. The single colony exhibiting this pattern was grown on rich liquid medium followed by growth on plates containing 5-fluoroorotic acid (5FOA) to counter-select cells that underwent a second recombination event (‘pop-out’, Figure 2A #6). Notably, only a minority of the ‘pop-out’ colonies obtained (2 out of 30 colonies analyzed), had the shorter amplicon size, indicative of a cured *polB* allele (Figure 2C), while the majority reverted to the w.t. state. This bias hints that either there is a recombination preference for the event reproducing the w.t. (intein-containing) allele, or there is a substantial fitness advantage in having an intein-containing *polB* allele, as was previously shown for an archaeal group I intron [9].

### Curing the intein does not have a detrimental effect on growth under lab conditions

The intein-cured strain obtained following the ‘pop-out’ recombination step (Figure 2C lane 3) was further characterized. The growth rate of this strain was examined in comparison to that of the parent strain WR532 under standard lab conditions (42°C, HY medium). As seen in figure 2D, no significant change in growth was observed, and similar results were obtained for 37°C and 45°C (data not shown). It therefore follows that the relative scarcity of cured ‘pop-out’ colonies cannot be due to some severe fitness disadvantage caused by loss of the intein. Nevertheless, it is possible that under some yet unknown condition, the presence of the intein could alter the fitness of *Hfx. volcanii*, since it has been shown in yeast that a HEN may also posses a different unrelated role, such as that of a transcription factor [10].

### *In vivo* activity of the HEN extends to plasmid-encoded recognition sites

In order to assess the homing efficiency and specificity, we constructed four plasmids containing partial *polB* segments (Table 1). Three plasmids contained the original intein target site, i.e. the intein-less *polB* sequence, with flanking regions of varying lengths, from 850 to 250 bp, and one plasmid contained 850bp long flanking regions and a mutated homing site with several non-synonymous substitutions. This plasmid was generated since it had been demonstrated *in vitro* that HENs can easily tolerate single synonymous substitutions, but not non-synonymous ones [11], [12]. Since homing is a gene conversion process, which requires homologous recombination, the length of flanking regions upstream and downstream of the cleavage sites should affect homing efficiency, should homologous recombination be the primary mechanism *in vivo*.

Reason suggested that since the chromosome-encoded HEN was highly active on chromosomal sites (see above), it should also cleave and gene-convert a plasmid-encoded target site.

**Figure 3:**
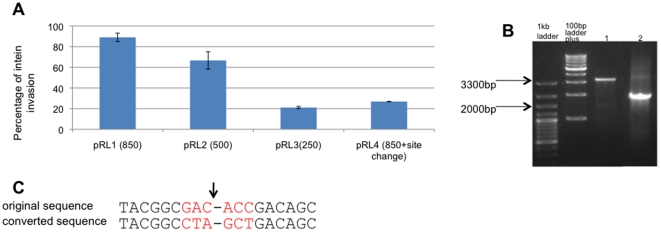
*In vivo* activity of the HEN extends to plasmid-located recognition sites. **A**. The percentage of intein invasion into four different constructs. The screen was performed by colony PCR and agarose gel electrophoresis. The numbers represent the average of two independent experiments. Bars represent standard error of the mean. **B**. Agarose gel electrophoresis of PCR amplicons from a colony transformed by pRL1, with primers RP2 and M13-F (located on the plasmid). Lane 1- a site invaded by an intein corresponding to a length of approximately 3.3 kb; lane 2- a vacant site of about 2kb. **C**. The non-synonymous substitutions engineered in pRL4, arrow indicates intein integration site.

The largest construct (pRL1) contains approximately 1700 bp of the *Hfx. volcanii polB* gene, providing flanking regions of approximately 850 bp at each side (Figure 3). Following transformation of pRL1 into *Hfx. volcanii*, transformant colonies were screened by colony PCR using primers M13-F and RP2 (Figure 3 and Table S1). Nearly 90% of colonies screened (44 out of 49 colonies analyzed) yielded a PCR product matching the size of the intein-containing *polB* allele. About 10% of the colonies displayed PCR products of a smaller size, corresponding to the original construct (Figure 3B and Table S2). Sequencing of selected plasmids from each category verified those findings.

Even single non-synonymous mutations in the target site dramatically reduce HEN activity [11], [12]. In a fourth construct (pRL4) the homing site was changed by introducing non-synonymous substitutions, one codon before the homing point and one codon after the homing site (Figure 3C). This change reduced intein homing to only 27% of the colonies, despite having flanking regions that were 850bp long on both sides, as in pRL1. This level of specificity again supports a homing mechanism rather than homing-independent gene conversion.

Flanking regions shortened to approximately 500 bp on each side of the target site (pRL2) reduced the recombination efficiency from 90% to 67% (16/24). Additional shortening of the flanking sequences to approximately 250 bp each (pRL3) further reduced the efficiency of homing to 20% (8/39) (Figure 3A). These results confirm that the homing process observed is mediated by the homologous recombination machinery of *Hfx. volcanii*, which requires longer stretches of highly similar sequences.

A previous study regarding an archaeal HEN of the DOD family, I-*DmoI* from the hyperthermophile archaeon *Desulfurococcus mobilis*, showed its *in vivo* activity when transformed into another archaeal species, *Sulfolobus acidocaldarius* [9]. In that study, the HEN was located in an rRNA intron. I-*DmoI* invaded an intron-less sequence when supplied on a suicide vector, either by elecroporation of the plasmid or by mating between neighboring *Sulfolobus* cells. Aagaard and coworkers also reported a fitness advantage for the cells containing the mobile intron. Interestingly, this was not the case in our study. Further work attempting to screen different growth media and environmental stresses for such a fitness effect on *Hfx. volcanii* should be performed.

Apart from its evolutionary importance, the *polB* HEN represents a highly valuable molecular genetic tool. *Hfx. volcanii*, serves as a genetic model organism for the domain of Archaea, [8], [13], [14] and has also recently become a target for DNA repair studies [15]. The existence of a highly efficient, specific endogenous endonuclease may facilitate the study of DNA double strand break repair, since the target sequence is specific, and does not exist elsewhere in the genome. Such a system has been very useful for studying DNA repair in yeast [16] and could help advance this field in the third, and sometimes neglected, domain of life.

## Materials and Methods

### Strains and culture conditions

The *Hfx. volcanii* strain used was WR532 (H26) *ΔpyrE2* [14]. Construction of the intein-cured strain (HAN12) is described in the results.

*Hfx. volcanii* was routinely grown in rich (HY) medium containing (per liter): 150 g of NaCl, 36.9 g of MgSO_4_ · 7H_2_O, 5 ml of a 1 M KCl solution, 1.8 ml of a 75-mg/liter MnCl_2_ solution, 5g yeast extract (Difco) and Tris-HCl (pH 7.2) at a final concentration of 50 mM. After autoclaving and cooling, 5 ml of 10% (w/v) CaCl_2_ were added. Agar plates contained 18 g of Bacto Agar (Difco) per liter. Casamino Acids (CA) medium contains the same components of the HY medium except that the yeast extract is replaced by 5 g/liter of Casamino acids (Difco).

For counter-selection of uracil auxotrophs, 5-fluoroorotic acid (5-FOA) (United States Biological) was added to the medium at a final concentration of 100 µg/ml. When required, uracil was added to a final concentration of 50 µg/ml.

### Transformation

Transformation of *Hfx. volcanii* was carried out using the PEG method as described in [17]. Briefly, 1.5 ml of liquid culture were grown to OD_600nm_ of 1.5, then centrifuged at 3500g for 5 minutes. The supernatant was discarded and the cells were resuspended in 200µl spheroplasting solution (1 M NaCl, 27 mM KCl, 50 mM Tris-HCl PH 8.2, 15% sucrose) and incubated at room temperature for 5 minutes. 20 µl of 0.5 M EDTA were added and cells were incubated at room temperature for 10 minutes. 10 µl of purified plasmid DNA were mixed with 15 µl spheroplasting solution and 5 µl of 0.5M EDTA were added to the cells, followed by incubation of 5 minutes at room temperature. Subsequently, 240 µl of PEG solution (60% PEG 600 inspheroplasting solution) was added and cells were incubated for 20 more minutes at room temperature. Following the incubation, 1 ml of regeneration solution (3.4M NaCl, 175mM MgSO4, 34mM KCl, 5mM CaCl2, 50mM Tris HCl pH 7.2, 15% sucrose) was added and cells were centrifuged at 3500 g for 7 minutes. The supernatant was discarded and cells were resuspended in HY medium supplemented with 15% sucrose and left to incubate without shaking overnight at 37°C. The cultures were then transferred to a 37°C shaker and left for an incubation of 3 more hours, then washed and plated on selective media.

### Gene knockouts

The gene knockouts was performed according to the protocol described in [7], [8]. In this method, the upstream and downstream flanking regions of the sequence to be exchanged are amplified by PCR and cloned together into the ‘suicide plasmid’ pTA131 that carries the *pyrE* selectable genetic marker and cannot replicate autonomously in *Hfx. volcanii*. The plasmids are then transformed into a *Hfx. volcanii ΔpyrE* mutant and transformants, in which the plasmids have been integrated into the chromosome, are selected for on plates that lack uracil (‘pop-in’). Upon counter-selection on plates containing uracil and 5-fluoroorotic acid (5FOA), the only cells that survive are those in which the integrated plasmids have been excised by spontaneous intra-chromosomal homologous recombination (‘pop-out’), either restoring the wild-type gene or resulting in allele exchange.

Curing of the intein was performed by allele exchange using the ‘pop-in’-‘pop-out’ methodology as described above. The intein-less sequence was generated by separately amplifying the upstream and downstream regions of the wild type *polB* intein, using primers that generate an overlap of approximately 15 nucleotides between the 3′ end of the upstream region and the 5′ end of the downstream region (for primers see Table S1). The two parts were assembled using overlap PCR to generate an intein-less polB construct. The ‘pop-in’ and ‘pop-out’ strains were screened using pairs of external ‘intein short-up’ (RP1) and ‘intein short down’ (RP2) primers located approximately 150bp upstream of the intein and 150bp downstream of the intein.

### Determination of intein presence on the exogenic target plasmids

The presence of an intein on the exogenic plasmids was tested by PCR using ‘intein short was conducted with the Phusion® DNA Polymerase (Finnzymes) according to the manufacturer's protocol.

### Determination of growth rates of the *w.t* and deletion strains

To compare the growth rates of the *w.t* strain and intein deletion strains, each strain was grown over-night in CA+uracil media at 42°C to the late log phase and then diluted to a fresh medium and left to shake at either 37°C, 42°C or 45°C. Turbidity of the culture (OD_595nm_) was measured every 3–5 hours using the Genesis 200 Workstation robot (Tecan).

### Plasmids and primers

A list of all plasmids that were used in this study is given in Table 1. A list of all primers used in this study is given in Table S1.

Plasmids intended for gene knockout had their inserts cloned between the *Hin*dIII- *Not*I restriction sites within the pTA131 multiple cloning site.

## Supporting Information

Figure S1 **Multiple sequence alignment of the PolB intein in different archaea.** Colors denote conserved functional blocks. Hvo- *Haloferax volcanii*, Hwa- *Haloquadratum walsbyi*, Ton- *Thermococcus onnurineus* Tzi- *Thermococcus zilligii.*
(PDF)

Figure S2 ***In vivo*** **homing into the integrated plasmid (‘pop-in’).** **A**. Agarose gel electrophoresis of PCR analysis on intein ‘pop-in’ candidates, using primers RP1 and RP2. All lanes ‘pop in’ with intein duplication see figure 2 A #3,4. **B**. Agarose gel electrophoresis of PCR analysis on intein ‘pop-in’ candidates, see figure 2A stage 5. I- using RP6 and M13R. II-using M13F and RP5. The different lanes signify different annealing temperature. **C**. Agarose gel electrophoresis of PCR analysis on intein ‘pop-in candidates, to examine ‘pop-in’ arrangement, see figure 2A stages 3 and 4. using primers RP4 and RP2 distinguishing between the two ‘pop-in’ arrangements. Lane 1- w.t.; lane 2 – intein ‘pop in’ corresponding to the arrangement seen in figure 2 stage 3. **D**. A schematic representation of the *polB* region, following ‘pop-in’. Arrows represent primer binding sites used in C.
(PPT)

Table S1 **Primers used in this study.**
(DOC)

Table S2 **Homing efficiencies for the different constructs per experiment.**
(DOC)
