# Supplementary material for: In Vivo Characterization of the Homing Endonuclease within the polB Gene in the Halophilic Archaeon Haloferax volcanii
Source: PLoS One. 2011 Jan 20;6(1):e15833. doi: 10.1371/journal.pone.0015833 (PMC3024317; doi:10.1371/journal.pone.0015833)
Supplement: Figure S1 — Multiple sequence alignment of the PolB intein in different archaea. Colors denote conserved functional blocks. Hvo- Haloferax volcanii, Hwa- Haloquadratum walsbyi, Ton- Thermococcus onnurineus Tzi- Thermococcus zilligii. (PDF) [file pone.0015833.s001.pdf]

Figure S1:

Block A, Block B, Block C, Block D, Block E, Block H, Block F, Block G

```
Hvo polB        DSVTGDRPVVVRDPGGTVRILPIEDLFARGTT-ESEVLIAADGDVVASATPGKTRRAL
Hwa PolB-3      DSVTGDRPVVVRDPSDYIQIVPIKLLFEQATAPEQNMRLTADGAPSVNSELPKERRHL
Ton-NA1 Pol     NSILPDEWVPLLIDG-RLKLTRIGDFVDNAMD-EGNPLKSNETEVLEVLGIN------
Tzi Pol         NSILPDEWIPLLING-RLKLVRIGDFVDSAMK-ELKPMKRDETEVLEVSGIG------

Hvo polB        DGWDALSVN-EDGEAEWQPIAQAIRH-NTDKPVVNLQHKFGES-TTTRDHSYVVPGED
Hwa PolB-3      DQWEALSLS-DTGETEWQPINQIIRH-QTDKEILTLQHEYGES-TTTRDHSYITADDG
Ton-NA1 Pol     ----AISFNRKTKISEVRPVRALIRH-RYRGKVYSIKLSSGRKIKVTEGHSLFTVKNG
Tzi Pol         ----AISFNRKTKRSETMPVRALLRH-RYSGKVYGIKLSSGRKIKVTAGHSLFTFRDG

Hvo polB        GLTTVSPDDVAE------PYRVS---------------GVPDVEPVEQV--------
Hwa PolB-3      EYVETSPENVDE------PLPIP---------------NIASVKTIETI--------
Ton-NA1 Pol     ELVEVTGGKVKPGDFIAVPRRINLPERHERINLADVLLNLPEEETADVVLTIPTKGR
Tzi Pol         ELVEIKGEEIKPGDFIAVPGRINLPERQERINLVEVLLGLPEEETADIVLTIPVKGR

Hvo polB        ----------------------------DVYEVLRGYEREYEDGRSVGSDNSITKRK
Hwa PolB-3      ----------------------------DIYQTL-TTDTQAQIGNDTEPDKWLPSAD
Ton-NA1 Pol     KNFFRGMLRTLRWIFEGEKRPRTARRYLEHLQKL-GYVRLKKIGYEVLDEKALRKYR
Tzi Pol         RNFFKGMLRTLRWIFGEEKRPGTARRYLEHLQTL-GYVRLGKIGYEIVNEEALRDYR

Hvo polB        QIHA-----------DDEYVWFG---HEHHRDVDSTVKVKRFVDIDSE--------D
Hwa PolB-3      CIHA-----------NDEYVWIGTTDKQQDRD-DSTPAIPRYIDLTSD--------T
Ton-NA1 Pol     ALYEVLAEKVRYNGNKREYL----VAFNDLRD------KIEFMPEEELREWKIGTLN
Tzi Pol         GLYETLTGKVKYNGNKREYL----VHFNDLRD------IIRLMPEKELKEWKVGTLN


Hvo polB        G----------AALIRLLGAYVPEGSASTGETAT--SKFGASLAESDREWLAQLQRD
Hwa PolB-3      G----------HALIRFLAVYLSDWSKSTITTTE--RGQCLHITGPQESALKTCAAD
Ton-NA1 Pol     GFRMEPFIEVNEDLAKLLGYYVSEGYAGKQRNQKNGWSYSVKLYNNDQKVLDDMERL
Tzi Pol         GFRMETSIEVKEDFAKLLSYYVSEGYAGKQRSQKNGWNYSVKLYNNDQNVLDDMETL

Hvo polB        YSRLFENTTAGIITSDRRAERTVEYQTDTGGASVTYNDETLKLQMMNELAAVFFREF
Hwa PolB-3      ADQLFTHITPSIAVDAESNTNTV-------------DSGFRCHIPTTLATTLISAF
Ton-NA1 Pol     ASKFFGKV--------RRGKNYVE--------------------MPKKMAYVLFKSL
Tzi Pol         ASKFFGKV--------RRGKNYVE--------------------IPRKMAYVLFESL

Hvo polB        AGQTSRGKRIPSFVFHLPEEKQDLFLTLLV------EGDGSREFPRYTEAYAQRNFD
Hwa PolB-3      AGHPAHTKQIPSIVYHLPAAEQSLFIRHLIQAESTPESDGVSGRPQKSDKPILLENE
Ton-NA1 Pol     CGTLAENKRVPEVIFTSPENVRWAFLEGYF------IGDG--------DLHPSKRVR
Tzi Pol         CGTLAENKRVPEIIFTSPESVRWAFLEGCF------IGDG--------DLHPGKGVR

Hvo polB        FETTSRELAAGLSMLLTQRGQKHSLKYRDSKDSYTIRTCST-------YR-------
Hwa PolB-3      FITTNRELAAGVSMLLTQCGQSYTISKQDTKGAYTIHINNS-------SS-------
Ton-NA1 Pol     LSTKSETLVNGLIILLNSLGIS-AVKIRFESGVYRVLVNEELSFLGNSKKKNAYYSH
Tzi Pol         LSTKSEELVNGLVILLNSLGVS-ALRIWLDSGVYRVLVNEELPFLDKGKKKTPY---

Hvo polB        --------------------------------------------EGRDPVLTEA--
```

```
Hwa PolB-3      ---------------------------------------------SGCTPTLTET--
Ton-NA1 Pol     VIPKEILEDVFEKRFQKNVSPKKLREKIKRGELNQEKAKRISWLLEG-DIVLDRVEE
Tzi Pol         VTSKEIPEEAFGKRFQRNISLEKLREKVEKGEPDAEKVKRVVWLLEG-DIVLDRVEE

Hvo polB        ----DHDGYVYDLSVEENENFVDGVGGIVLHNT
Hwa PolB-3      ----THSGYVYDLSVATNQNFVDGLGGLVLHNT
Ton-NA1 Pol     VEVEDYNGYVYDLSVEENENFLAGFGMIYAHNS
Tzi Pol         VAVDDYEGYVYDLSVEENENFLAGFGMLYAHNS
```
