# Supplementary material for: In Vivo Characterization of the Homing Endonuclease within the polB Gene in the Halophilic Archaeon Haloferax volcanii
Source: PLoS One. 2011 Jan 20;6(1):e15833. doi: 10.1371/journal.pone.0015833 (PMC3024317; doi:10.1371/journal.pone.0015833)

## Slide 1
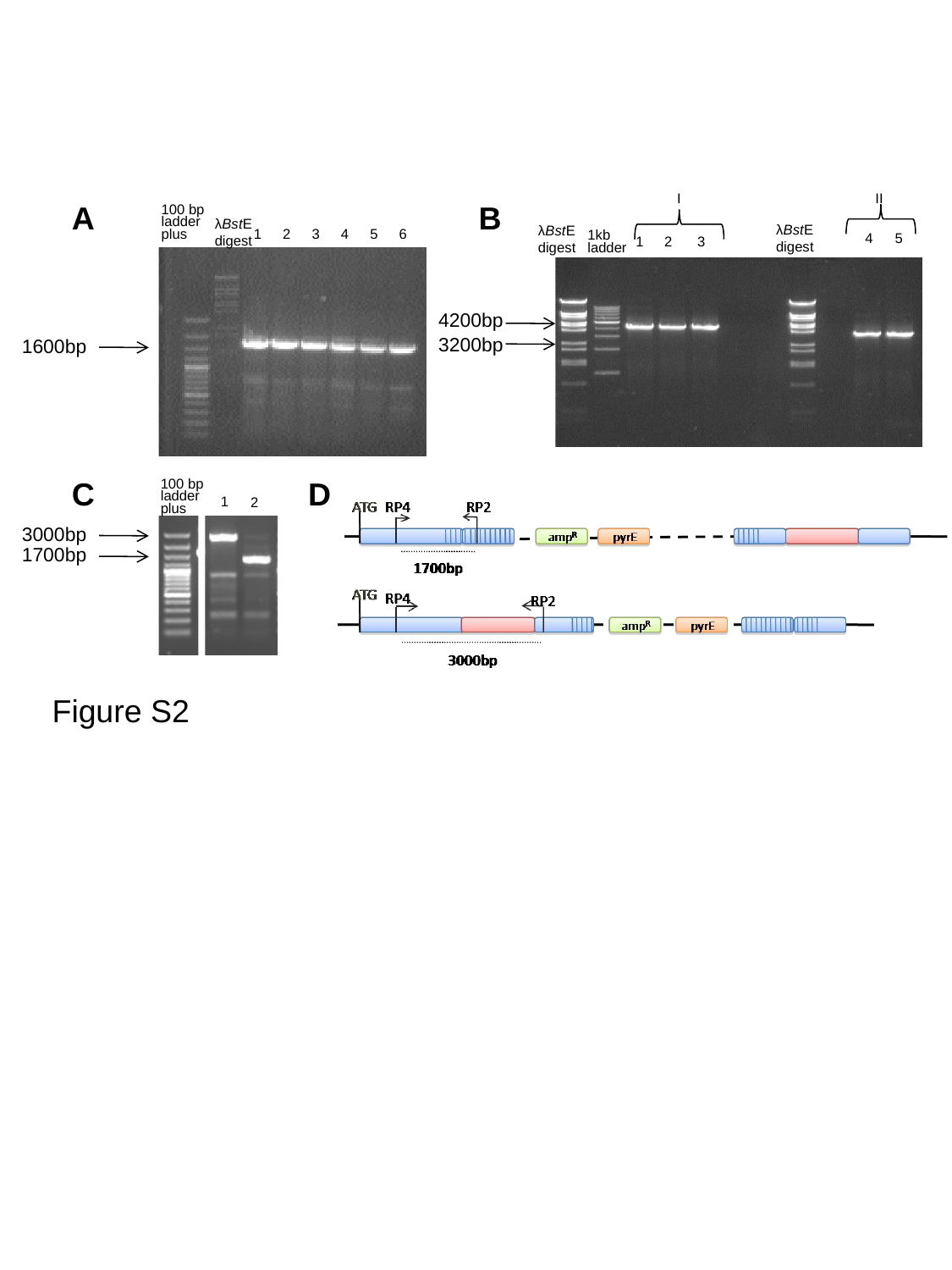

I
II
A
B
100 bp ladder plus
λBstE digest
λBstE digest
λBstE digest
1
2
3
4
5
6
4
5
1kb ladder
1
2
3
4200bp
3200bp
1600bp
C
D
100 bp ladder plus
1
2
3000bp
1700bp
Figure S2

## Slide 2
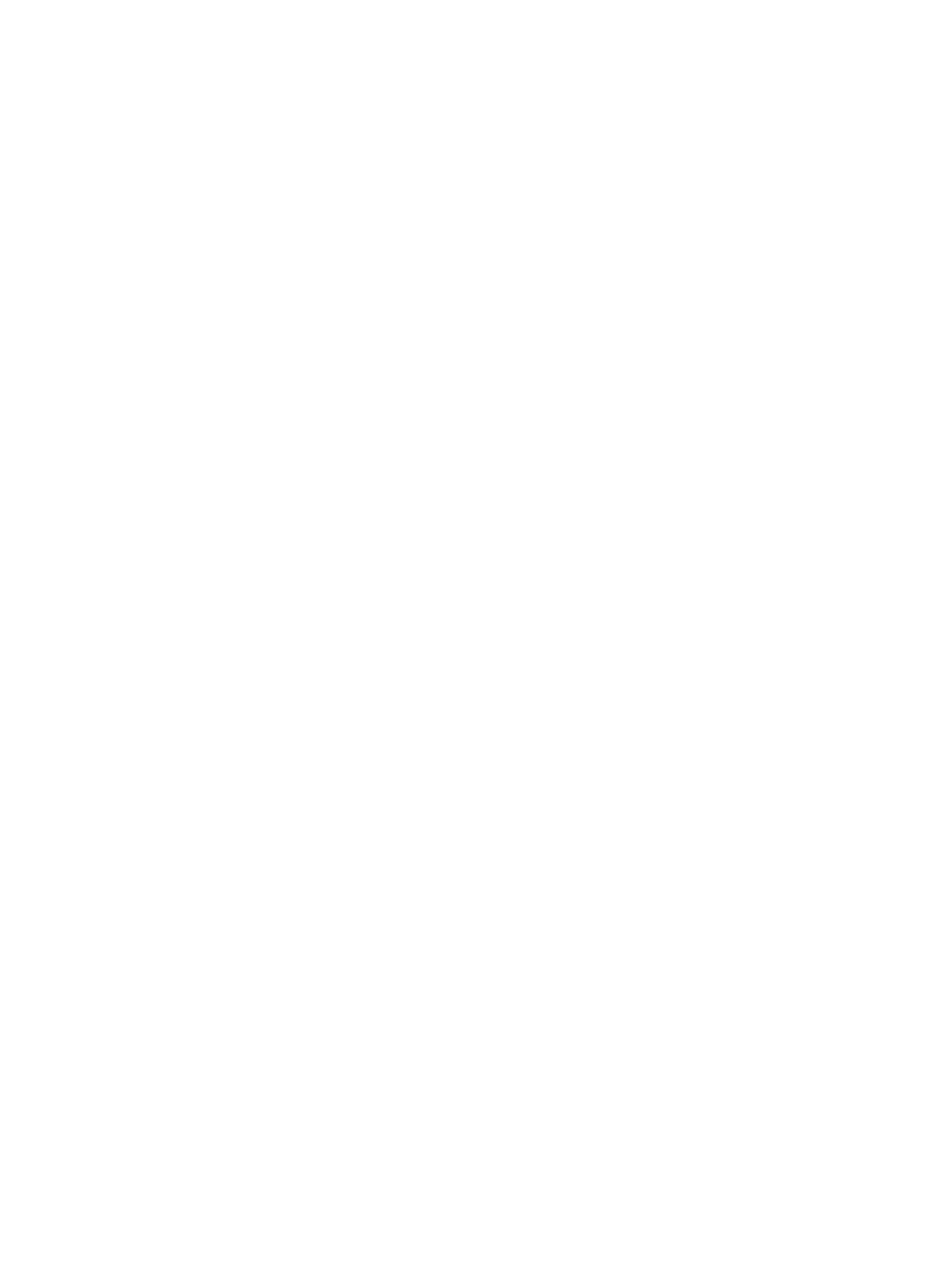

Supplement: Figure S2 — In vivo homing into the integrated plasmid (‘pop-in’). A. Agarose gel electrophoresis of PCR analysis on intein ‘pop-in’ candidates, using primers RP1 and RP2. All lanes ‘pop in’ with intein duplication see figure 2 A #3,4. B. Agarose gel electrophoresis of PCR analysis on intein ‘pop-in’ candidates, see figure 2A stage 5. I- using RP6 and M13R. II-using M13F and RP5. The different lanes signify different annealing temperature. C. Agarose gel electrophoresis of PCR analysis on intein ‘pop-in candidates, to examine ‘pop-in’ arrangement, see figure 2A stages 3 and 4. using primers RP4 and RP2 distinguishing between the two ‘pop-in’ arrangements. Lane 1- w.t.; lane 2 – intein ‘pop in’ corresponding to the arrangement seen in figure 2 stage 3. D. A schematic representation of the polB region, following ‘pop-in’. Arrows represent primer binding sites used in C. (PPT) [file pone.0015833.s002.ppt]
