# Supplementary material for: In Vivo Characterization of the Homing Endonuclease within the polB Gene in the Halophilic Archaeon Haloferax volcanii
Source: PLoS One. 2011 Jan 20;6(1):e15833. doi: 10.1371/journal.pone.0015833 (PMC3024317; doi:10.1371/journal.pone.0015833)
Supplement: Table S1 — Primers used in this study. (DOC) [file pone.0015833.s003.doc]

**Table S1.**

| **Primer** | **Sequence (5' to 3')** | Used for | Location on polB |
| --- | --- | --- | --- |
| **AP58**-int-del up5 HindIII | AAGCTTGCGGCTCGGCGAGGTGTG | Construction of pAN9 | 978-F |
| **AP59**-int-del up3 flankings | GACGCTGTCGGTGTCGCCGTAGGCCACGTCGTA | Construction of pAN9 | 3189-R |
| **AP60**-int-del down5 flankings | CTACGGCGACACCGACAGCGTCATGCTCG | Construction of pAN9 | 1866-F |
| **AP61-**int-del down3 NotI | GCGGCCGCCGGTGCCGTCGTCTATGTAA | Construction of pAN9 and pAN14 | 35bp after the stop codon -R |
| **RP1**-intein short up | TCTAGACGTGCTGGGCTGGGACCG | Screening of the pop-in pop-out. | 1743-F |
| **RP2**-intein short down | AAGCTTCGCCGGTACAGCTTCTCGAA | Screening of the pop-in pop-out, and the existence of an intein on the exogenic plasmids. | 3337-R |
| **AP8** | AAGCTTCCTCGAAGGGGAAACAGGA | Sequencing of the pAN9 pop in cloned to pGEM | 1378-F |
| **AP9** | TCTAGAGCCGTAGGCCACGTCGTA | Sequencing of the pAN9 pop in cloned to pGEM | 1855-R |
| **AP10** | TCTAGAGACAGCGTCATGCTCGAAC | Sequencing of the pAN9 pop in cloned to pGEM | 3191-F |
| **AP12** | GGGACGCCCTCTCTGTG | Sequencing of the pAN9 pop in cloned to pGEM | 2051-F |
| **AP15** | CAGGTCGTACACGTAGCCG | Sequencing of the pAN9 pop in cloned to pGEM | 3111-R |
| **AP87** | GGGCGCGACTTCCTGGTGA | Sequencing of the pAN9 pop in cloned to pGEM | 564-R |
| **AP41** | GCTTGCGGCCCGGACATCAAGG | Sequencing of the pAN9 pop in cloned to pGEM | 1019-F |
| **RP4** | GCGCCCCGACACGTTGCCGA | Sequencing of the pAN9 pop in cloned to pGEM | 700-F |
| **RP5** | CGTAGACTTGCCGGACCCGTTGG | Sequencing of the pAN9 pop in cloned to pGEM | 353bp after stop codon -R |
| **RP6** | AGCCGCGCCGCCGAAGC | Sequencing of the pAN9 pop in cloned to pGEM | 58-R |
| **RP7**-polB1000-F ClaI | ATCGATAAGCTCGAAGACGCG | Construction of pRL2 | 1293-F |
| **RP8**- polB1000-R-NotI | GCGGCCGCCGAGCCCCGCCCGAA | Construction of pRL2 | 3703-R |
| **RP19-**polB 500-F-ClaI | ATCGATCGGGAGGAAAAGAAGTCGCTGCG | Construction of pRL3 | 1633-F |
| **RP20-**polB 500-R-NotI | GCGGCCGCGAAGCCCGTGATGTCGATGTCGTC | Construction of pRL3 | 3424-R |
| **RP9** | ATCGATGCGGCTCGGCGAGGTGTG | Construction of pRL4 | 984-F |
| **RP10** | GCGGCCGCCGGTGCCGTCGTCTATGTA | Construction of pRL4 | 35bp after the stop codon -R |
| **RP11** | CTAGCTGACAGCGTCATGCTCGAACTCGGC | Construction of pRL4 | 1866-F |
| **RP12** | GCTGTCAGCTAGGCCGTAGGCCACGTCGTA | Construction of pRL4 | 3189R |
| **M13F** | GTTTTCCCAGTCACGACGTTG | Located on pTA131, used to examine the pop-in state |  |
| **M13R** | AACAGCTATGACCATGATTACG | Located on pTA131 used to examine the pop-in state |  |
