# Supplementary material for: In Vivo Characterization of the Homing Endonuclease within the polB Gene in the Halophilic Archaeon Haloferax volcanii
Source: PLoS One. 2011 Jan 20;6(1):e15833. doi: 10.1371/journal.pone.0015833 (PMC3024317; doi:10.1371/journal.pone.0015833)
Supplement: Table S2 — Homing efficiencies for the different constructs per experiment. (DOC) [file pone.0015833.s004.doc]

**Table S2.**

|  | pRL1 | pRL2 | pRL3 | pRL4 |
| --- | --- | --- | --- | --- |
| Intein invasion (total colonies tested) 1^st^ experiment | 27*(29) | 9(12) | 9(12) | 4(15) |
| Percentage of intein invasion, 1^st^ experiment | 93.1% | 75% | 22.2% | 26.67% |
| Intein invasion (total colonies tested) 2^nd^ experiment | 17(20) | 7(12) | 6(30) | 13(48) |
| Percentage of intein invasion, 2^nd^ experiment | 85% | 58.3% | 20% | 27.1% |
| Average percentage of intein invasion in both experiments | 89.05% | 66.67 | 21.1 | 26.88 |

n=2

*5 colonies showed mixed results (both an invasion and a vacant site), the plasmids were extracted transformed into *E. coli* cells, and 90% of them were contained an intein. Thus, all these colonies were counted as containing an intein invasion.
